# Evaluation of flavonoid composition and biological activities of hydrolyzed whole pomelo juice

**DOI:** 10.3389/fnut.2025.1730735

**Published:** 2026-01-07

**Authors:** Xi Xie, Shanshan Zhuang, Yanxia Gu, Yunrui Shen, Weisi Zhang, Lukai Ma, Gengsheng Xiao, Qin Wang, Yonghui Zhong, Huifan Liu

**Affiliations:** 1 Key Laboratory of Green Processing and Intelligent Manufacturing of Lingnan Specialty Food of Ministry and Rural Affairs, College of Light Industry and Food, Zhongkai University of Agriculture and Engineering, Guangzhou, China; 2 Guangdong Provincial Key Laboratory of Lingnan Specialty Food Science and Technology, Zhongkai University of Agriculture and Engineering, Guangzhou, China; 3 Institute of Agricultural Sciences, Meizhou, Guangdong, China; 4 Guangdong Dayue Mingzhu Agricultural Technology Co., Ltd., Meizhou, Guangdong, China; 5 Guangdong Meizhou Treasure Golden Pomelo Industry Co., Ltd., Meizhou, Guangdong, China

**Keywords:** whole pomelo juice, flavonoids, anti-inflammatory, antitussive, expectorant, activity

## Abstract

This study aimed to investigate the bioactive compounds and evaluate the antioxidant and anti-inflammatory properties of juice prepared from whole pomelo fruits (WPJ). Methods: Total flavonoid content, naringin levels, and nutrient composition of WPJ were quantified over the storage period. Flavonoid profiling was conducted using LC–MS. Anti-inflammatory activity was assessed in vitro using LPS-stimulated RAW264.7 macrophages, where dose–response effects on nitric oxide (NO), interleukin-6 (IL-6), and tumor necrosis factor-alpha (TNF-α) were measured. An in vivo phenol red–induced mouse model was used to evaluate the effects of WPJ on respiratory secretion and pulmonary inflammation, with analysis of MAPK/NF-κB signaling pathways. Results: WPJ exhibited a high total flavonoid content (950.68 ± 7.65 mg/100 g), dominated by hesperidin, tribuloside, baicalin, apigenin 7-O-neohesperidoside, hesperetin, neohesperidin dihydrochalcone, naringenin, naringin, jaceosidin, and pinocembrin. In vitro, WPJ pretreatment significantly reduced NO, IL-6, and TNF-α production in LPS-stimulated RAW264.7 cells in a dose-dependent manner. In vivo, WPJ promoted phenol red secretion in the respiratory tract and attenuated pulmonary inflammatory responses, partly through inhibition of MAPK/NF-κB signaling. Conclusion: The results demonstrate that WPJ is rich in diverse flavonoids and possesses potent anti-inflammatory activity both in vitro and in vivo. Its ability to suppress key inflammatory mediators and modulate respiratory tract responses suggests potential benefits for pulmonary health. These findings support the potential application of WPJ as a functional food for preventing or alleviating cough- and phlegm-related conditions.

## 1 Introduction

Nowadays, functional beverages have gained significant popularity due to their incorporation of nutraceutical compounds, including vitamins, minerals, antioxidants and other bioactive compounds, which are believed to promote health and may reduce the risk of chronic diseases (72). Citrus fruits are widely recognized with their distinct flavor and high nutritional value, making them suitable for both fresh consumption and processing products including juice and puree (73). However, traditional processing methods generate substantial waste, including peels, pulp, and seeds. Therefore, utilizing the entire fruit can improve the overall utilization rate of citrus fruits and enhance the added value of the citrus industry.

Pomelo is a member of the Rutaceae family belonging to Citrus fruits. It is widely cultivated in southern China and the fruits contains a number of nutrients that are beneficial to human health. Recent phytochemical studies have indicated that flavonoids (1), dietary fiber (2, 3), and pectin (4) are the main chemical constituents of pomelo. Pharmacological studies have found that pomelo flavonoids possess several activities, such as antioxidant (5), anti-tumor (2, 3), antidiabetic (6), and anti-inflammatory effects (7) as well as having beneficial effects on atherosclerosis (8). Pomelos are predominantly consumed as fresh fruit, while processed products account for only a small proportion of their overall utilization. In addition, previous studies investigated the functions and applications of flavonoids in pomelo, however, have predominately focused on its peel, but the whole fruit functions remain unclear (9–12). The processing and utilization of pomelo resources play a critical role in minimizing waste and enhancing their overall utilization. Therefore, comprehensive utilization of the entire pomelo fruit may significantly enhance resource efficiency and serve as a sustainable source of raw materials for the development of various value-added products.

Enzymatic hydrolysis involves the use of specific enzymes to hydrolyze macromolecules, resulting in breaking down them into simpler compounds. For example, the cell walls of fruit predominantly consist of cellulose, hemicellulose, and pectin, which are the insoluble components, making the juice unstable (74). The use of cellulase, hemicellulase, and pectinase can degrade the structures within fruit cell walls, releasing nutrients and bioactive compounds, while also breaking down larger pellets into smaller particles in the juice (13–15). Many studies showed that after enzymatic hydrolysis, the mineral elements, total amino acid, vitamin C, and organic acid content of fruit juice increased (16, 17). Pomelo are rich in nutrients such as polyphenols, organic acids minerals and dietary fiber. However, limited attention has been paid to the production of whole pomelo juice (WPJ) via enzymatic hydrolysis, and its nutraceutical properties remain largely uncertain.

Coughing is a defensive reflex of the body that clears secretions and harmful factors from the respiratory tract (18), and may be caused by several respiratory diseases. Clinically, it is characterized by the chronic process of coughing up phlegm, wheezing, and recurrent attacks (19). Inflammatory responses are involved in the pathogenesis of various respiratory disorders (20). However, most of the currently available antitussive drugs are associated with significant side effects. Therefore, there is a pressing need to investigate natural medicines and their active ingredients that can effectively relieve coughing and promote the resolution of phlegm (21), For example, Dong et al. prepared naringenin nanosuspensions, which improved the bioavailability of naringenin and enhanced expectorant effects (22). Ge et al. confirmed that a flavonoid from *Elaeagnus angustifolia* had pharmacological activities, and could be utilized in the treatment of asthma and chronic bronchitis (23). While pomelo is used as an effective fruit to relieve coughing in folk medicine; only a few preliminary studies have been conducted to investigate the cough relieving effect of the whole pomelo (24). In this study, we first processed pomelo by juicing and enzymic hydrolysis, then conducted a shelf-life test. The nutritional content and flavonoids were then extracted for characterization. In addition, *in vivo* expectorant and antitussive effects of WPJ were further evaluated for confirming its function on prevent cough and phlegm related diseases.

## 2 Materials and methods

### 2.1 Materials and reagents

Pomelo was obtained from Meizhou Zhenbao Co., Ltd. (Guangdong, China). RAW 264.7 cells were procured from the Type Culture Collection of the Chinese Academy of Sciences (Shanghai, China). Lipopolysaccharide (LPS) and Griess reagent were obtained from Sigma-Aldrich (St. Louis, MO, United States). Mouse TNF-*α*, IL-1β, IL-6, and IL-10 detection enzyme-linked immunosorbent assay (ELISA) kits were obtained from Jiancheng Biotechnology Co., Ltd. (Nanjing, China). Transwell plates (12-well, polyester film, aperture 0.4 μm, membrane area 1.12 cm^2^) were obtained from Corning Inc. (Somerville, United States). The 3-(4,5-dimethylthiazol-2-yl)-2,5-diphenyl tetrazolium bromide (MTT) cell proliferation and toxicity test kit was purchased from Bebo Biotechnology Co. Ltd. (Guangzhou, China). RIPA (strong) tissue cell rapid lysate and the BCA protein concentration determination Kit were obtained from Solarbio (Beijing, China). The Kechuanning pill (OTC) was purchased from Jiangxi Durenhe Pharmaceutical Co., Ltd. (Jiangxi, China). All other chemicals and reagents were used in analytical grade.

### 2.2 Whole pomelo juice preparation

Fresh pomelos were selected and cleaned. Their peel and pulp samples were broken and pulped separately, and followed by enzymatic hydrolysis with pectinase and protease (0.3 g/100 g) mixture at 50–55 °C, 70–110 min (25). After enzymatic hydrolysis, the samples were degassed, homogenized, and then sterilized at 135 °C for 6 s, and the resulting product was the WPJ.

### 2.3 Shelf-life assessments

The WPJ was stored at 20–22 °C. To select the best active substance preservation stage for WPJ, the total flavonoid and naringin contents of the WPJ stored for 1, 3, and 5 years were determined to identify the optimal shelf life.

### 2.4 WPJ nutrient composition

The nutritional components of WPJ were analyzed. For total polyphenol determination, 20 μL of either the polyphenol standard or sample, 1,580 μL of distilled water, 100 μL of Folin–Ciocalteu reagent, and 300 μL of Na₂CO₃ (200 g/L) were added to a glass tube. The mixture incubated at 40 °C for 30 min in a water bath. Absorbance was then measured at 765 nm against a blank solution containing 20 μL of distilled water instead of the sample or standard, using a UV–Vis spectrophotometer (LAMBDA 650, CA, United States). For A total of 800 μL of distilled water, 200 μL of the polyphenol standard or sample, and 60 μL of NaNO₂ (5%) were added to a glass cuvette. After 5 min, 60 μL of AlCl₃ (10%) were added, followed by the addition of 400 μL of NaOH (1 mol/L) and 480 μL of distilled water after an additional minute. The resulting mixture was vortexed thoroughly, and the final reaction volume in the cuvette was adjusted to 2 mL. Absorbance was measured at 510 nm using a spectrophotometer (26). The quantification of naringin was carried out using high-performance liquid chromatography (HPLC), Juice extracted from grapefruits (fresh-pressed) was centrifuged at 8000 rpm for 15 min and the supernatant filtered through a number 1 Whatman filter and diluted 1:8 (v:v) with sodium acetate buffer 0.02 M, pH 4.0, before HPLC analysis (27). Vitamins B1, B2, B6, C, and E were quantified by HPLC. Vitamins were analyzed using a DAD detector at the following wavelengths: 240 nm for vitamins B1 and B12, 254 nm for vitamins B3, B6, and C, and 274 nm for vitamin B2, following the method described by Baranowska et al. (28). The contents of calcium, iron, potassium, magnesium, phosphorus, and zinc were determined using inductively coupled plasma atomic emission spectrometry (ICP-AES), following the method described by Santos et al. (29).

### 2.5 Structural analysis of WPJ flavonoids

#### 2.5.1 LC–MS measurement

Crude flavonoids were extracted from WPJ using the method described by Wang et al. (30). A 60% ethanol solution was employed as the solvent for ultrasonic–microwave co-extraction. The extraction was conducted at a controlled temperature of 60 ± 2 °C, and the supernatant was collected by centrifugation at 4000 × g for 10 min. The resulting samples were concentrated under vacuum and freeze-dried to yield crude flavonoid compounds, which were subsequently analyzed using liquid chromatography–mass spectrometry (LC–MS) for untargeted metabolite profiling. Liquid chromatography (LC) separation was performed on an ACQUITY UPLC® BEH C18 column (2.1 × 100 mm, 1.7 μm), using mobile phase A (0.1% formic acid in high-purity water) and mobile phase B (0.1% formic acid in acetonitrile). The gradient elution program was set as follows: 0–1 min, 20% B; 1–9 min, 20–50% B; 9–12 min, 50–98% B; 12–13.5 min, 98% B; 13.5–14 min, 98–20% B; and 14–17 min, 20% B. The ultraviolet (UV) detection wavelength range was 200–400 nm. Mass spectrometric detection was performed using an electrospray ionization (ESI) source operated in both positive and negative ionization modes. The spray voltages were set to 3.50 kV for the positive mode and 2.50 kV for the negative mode. The sheath and auxiliary gas pressures were 30 arb and 10 arb, respectively. The capillary temperature was maintained at 325 °C. Full-scan mass spectra were acquired at a resolution of 70,000 over an m/z range of 150–1,000. Tandem mass spectrometry (MS/MS) was conducted using higher-energy collisional dissociation (HCD) at collision energies of 10, 50, and 60 eV.

#### 2.5.2 Determination of the antioxidant properties of the flavone substances

With LC–MS identification, the ten most abundant flavonoids were selected for antioxidant capacity assessment. The radical scavenging abilities of these flavonoids against DPPH and ABTS were evaluated according to the method described by Zhuang et al. (31). Additionally, the protective effects of the ten flavonoids on AAPH-induced red blood cell hemolysis were investigated using the method described by Wang et al. (30). The tested sample concentrations ranged from 10 to 100 μg/mL.

### 2.6 Evaluation of cytotoxic, anti-inflammatory, and antioxidant activities

#### 2.6.1 Cell culture

The murine macrophage cell line RAW 264.7 was incubated in a humidified atmosphere with 5% CO_2_ at 37 °C. Dulbecco’s Modified Eagle’s Medium (DMEM, ILT, Carlsbad, CA, United States) contained 10% heat-inactivated fetal bovine serum, 100 μg/mL streptomycin, and 100 μg/mL penicillin was used for cell culture.

#### 2.6.2 Cell viability, nitric oxide and pro-inflammatory cytokines assessment

Cell viability was assessed using the MTT assay. Following removal of the cell supernatant, cell viability was evaluated using the MTT assay, as described by Soha et al. (32). The blank control (CK) group cultured with LPS-free culture medium, while the negative model group was stimulated with 1 μg/mL of LPS. Experimental groups were treated with varying concentrations of WPJ (25, 50, 75, and 95%) or Kechuanning (KCN, OTC; 5 g/kg). Kechuanning, a traditional Chinese medicine, has demonstrated significant efficacy in the treatment of asthma (33, 34). After 2 h treatment, cells were stimulated with 1 μg/mL of LPS. After an additional 24 h incubation, cell supernatants were collected by centrifugation (1,000 × g, 10 min), and levels of NO, TNF-*α*, IL-1β, IL-6, and IL-10 were quantified using Griess reagent and ELISA kits (Jiancheng Bioengineering Institute, Jiangsu, China).

### 2.7 Animals and administration

Male BALB/c-nu nude mice (specific pathogen-free grade, 4 weeks old), provided by Wuhan Hualianke Biotechnology Co. Ltd. (SYXK2018-0104, Hubei, China), were acclimatized to the laboratory conditions for 1 week before use. The breeding condition was 22–26 °C, 50–60% relative humidity, with artificial light for 12 h per day. After a one-week acclimation period, mice were randomly assigned to six groups of ten animals each: (1) a CK group (normal saline, administered orally at 15 mL/kg of body weight), (2) an ammonium hydroxide (NH₄OH) model group (distilled water, administered orally at 15 mL/kg of body weight; NH₄OH-induced), (3) a 25% WPJ group (low dosage, orally administered at 15 mL/kg with 25% WPJ of body weight; NH₄OH-induced), (4) a 50% WPJ group (medium dosage, orally administered at 15 mL/kg with 50% WPJ of body weight; NH₄OH-induced), (5) a 75% WPJ group (low dosage, orally administered at 15 mL/kg with 25% WPJ of body weight; NH₄OH-induced), and (6) a Kechuanning (KCN) group (positive control, orally administered at 5 g/kg with KCN of body weight; NH₄OH-induced).

Coughing was induced by exposing mice to NH₄OH. Before the experiment, mice were fasted for 12 h. Subsequently, each group of mice administered orally dose of 15 mL/kg of body weight, while the CK group received an equivalent volume of normal saline. One-hour post-administration, the mice were placed in a glass jar (10 × 10 × 10 cm) and exposed the vapor of 0.3 mL of 25% NH₄OH for 45 s. Afterward, the mice were removed to observe for any coughing responses. The number of cough responses was recorded over a 6 min period following NH₄OH exposure, using video monitoring for quantification. Mice in the experimental group were intraperitoneally injected with a 0.0125% (w/v) phenol red solution. After 30 min, a mouse model of the phlegm turbidity and lung obstruction was established. All laboratory animals were treated according to the national regulations on the usage and welfare of laboratory animals and were approved by the Institutional Animal Care and Use Committee, China [Reference No: SYXK2024].

### 2.8 Expectorant and antitussive assay

Phenol red secretion experiments were conducted to evaluate WPJ expectorant activity (35). The mice were randomly divided into groups of 10 and orally administered with the WPJ and KCN (over-the-counter drug, OTC) for 3 days.

After the final administration, 2.5% phenol red solution (0.2 mL) was intraperitoneally injected. Then, 30 min after the application of phenol red, the mice were sacrificed. Trachea was dissected and immediately placed into 1 mL of normal saline. After the trachea was washed, 0.1 mL of 1 M NaOH was added to the saline and the optical density was measured at 548 nm using a microplate reader (Thermo Fisher Scientific, CA, United States). Data were expressed as a percentage of the optical density of each experimental sample compared to that of the model control (78). The expectorant activities were assessed by the increase of the optical density in terms of that in model groups by Equation 1:


(1) $$\text{The percentage of increase}=[(D_{t}-D_{0})/D_{0}\times 100\%]$$

D_0_: the optical density of negative control, D_t_: the optical density of the experimental group.

The antitussive activities were expressed as the percentage of inhibition of the number of coughs in terms of that in model groups by using the Equation 2:


(2) $$\text{The percentage of inhibition}=[(C_{0}-C_{t})/C_{0}\times 100\%]$$

C_0_: the number of coughs of the model group, C_t_: the number of coughs of the experimental group.

### 2.9 Histopathological analysis

Liver pathology was evaluated using hematoxylin and eosin (HE) staining. First, the liver tissues were fixed in formalin solution (10%) and dehydrated with alcohol. The fixed tissues were then embedded in paraffin. Finally, 3-μm sections were stained with hematoxylin and eosin. The sections were observed at 200 × magnification and photographed (Leica Microsystems DM1000).

### 2.10 Inflammatory cell content

Whole blood mixed with fresh EDTA-K2 anticoagulant tubes was analyzed using an automatic blood cell analyzer (Mindray, BC-5380), and the inflammatory cell content (white blood cells, neutrophils, lymphocytes, monocytes, eosinophils, and basophilic) was detected.

### 2.11 Effects of on the inflammatory factors

A puncture needle was inserted into the upper end of the trachea, and the left lung was irrigated with PBS (2 mL) 3 times. The recovered alveolar lavage fluid was centrifuged at 4 °C for 10 min at 1500 g. The supernatant was collected for biochemical detection of the NO, IL-4, IL-6, and IL-10 using ELISA kits Jiancheng Bioengineering Institute (Jiangsu, China) following the manufacturer’s instructions. The middle lobe of the right lung was precisely weighed, and a 10% (w/v) lung tissue homogenate was prepared in an ice bath using normal saline, based on the weight-to-volume ratio. The homogenate was then centrifuged at 3,000 × g for 10 min, and the supernatant was collected. The concentration of TNF-*α* was subsequently determined using ELISA kits.

### 2.12 Western blotting

According to previous studies (36), the total protein of the liver tissue was extracted and detected using western blotting.

### 2.13 Data processing and analysis

All experimental results are expressed as the mean ± standard deviation (SD). Data were analyzed using one-way ANOVA tests in SPSS v19.0 (IBM, United States). Charts were prepared using Origin 2018 software (Origin Lab, United States).

## 3 Results and discussion

### 3.1 Determination of the best storage period

As shown in Supplementary Figure S1, the total flavonoid and naringin contents in WPJ decreased significantly over the storage period (*p* < 0.05). The total flavonoid content declined from 950.68 mg/100 g to 640.42 mg/100 g after 2 years of storage and further decreased to 600.19 mg/100 g after 5 years. Naringin, a major flavonoid in pomelo, was also quantified. Similar to the total flavonoid content, the naringin level decreased from 23.08 mg/L to 10.65 mg/L over 5 years of storage. As reported in previous studies, the stability of flavonoids is strongly affected by oxygen exposure, storage temperature, light conditions, and storage duration (36, 37). Therefore, the changes in nutritional compounds, particularly flavonoids, were compared across different storage periods. The one-year storage sample retained a relatively high flavonoid level; thus, WPJ stored for 1 year was selected for subsequent experiments.

### 3.2 Nutritional composition of the WPJ

Pomelo nutrients may be lost as they are processed into by-products, considering the comprehensive situation, we performed nutrient composition determination of the WPJ stored for 1 year, and the results are shown in Table 1. The total flavonoid content was 950.68 ± 7.65 mg/100 g, and this is an important source of the bitter taste in WPJ. WPJ contains calcium, iron, magnesium, potassium, phosphorus, and other minerals, among which the concentrations of calcium and potassium are highest at 121.12 ± 0.57 mg/L and 579.20 ± 0.16 mg/L, respectively. Pomelo is rich in vitamins. After 1 year of storage, the vitamin C content in the WPJ was 104.90 ± 0.28 mg/100 g and the niacin content was 230.53 ± 4.51 μg/100 g, indicating the nutrient components of the WPJ were not notably altered for 1 year of storage. In addition, the WPJ also contain a number of essential oils, soluble fiber and carbohydrate (Supplementary Table S1).

**Table 1:** Nutritional composition of WPJ.

Nutrients	Content
Polyphenols (mg/100 g)	35.60 ± 0.22
Flavonoids (mg/100 g)	950.68 ± 7.65
Naringin (mg/L)	23.08 ± 0.56
β-carotene (mg/100 g)	0.12 ± 0.01
Ca (mg/L)	121.12 ± 0.57
Cu (mg/L)	0.07 ± 0.01
Fe (mg/L)	2.31 ± 0.02
K (mg/L)	579.20 ± 0.16
Mg (mg/L)	41.07 ± 0.03
P (mg/L)	50.35 ± 0.37
Zn (mg/L)	0.48 ± 0.01
Vitamin B1 (mg/100 g)	0.64 ± 0.02
Vitamin B2 (mg/100 g)	0.53 ± 0.02
Vitamin B6 (mg/100 g)	0.32 ± 0.01
Vitamin E (mg/100 g)	52.41 ± 0.45
Vitamin C (mg/100 g)	104.90 ± 0.28
Niacin (ug/100 g)	230.53 ± 4.51

### 3.3 Structural characterization of the WPJ flavonoids

As the analysis, 679 substances of the WPJ were identified, of which 515 were not matched to entries in the KEGG database and were thus classified as unknown. However, chemotaxonomic classification revealed seven biologically active flavonoids from WPJ. Among the 138 detected metabolites, 26 were further classified using chemotaxonomic criteria. Integration of mass spectrometry and chemotaxonomic analysis identified a total of 31 bioactive flavonoid compounds in WPJ. These included 15 flavones/flavanones, 4 flavonols/flavanonols, 1 isoflavone/isoflavanone, 1 anthocyanin, 1 chalcone/dihydrochalcone, 2 chromones, and 9 other types (Table 2). Previous studies have demonstrated that flavones (38), flavonols (39), isoflavones (40), anthocyanins (41), chalcones (42), and chromones exhibit both antioxidant and anti-inflammatory properties. However, due to the complexity of flavonoid composition in WPJ, it remains unclear which specific compounds play key functional roles. Therefore, ten flavonoids present in high concentrations in WPJ were selected for antioxidant activity evaluation.

**Table 2:** Chemical identification table of flavonoids extract from WPJ.

Name	Formula	rt	*m/z*	ppm	Chemotaxonomic
Hesperidin	C_28_H_34_O_15_	842.04	610.18	16.98 ± 1.23	Flavones/Flavanones
Tribuloside	C_30_H_26_O_13_	936.87	577.12	14.59 ± 2.65	Flavonols/Flavanonols
Baicalin	C_21_H_18_O_11_	1015.98	429.08	11.51 ± 0.78	
Apigenin 7-O-neohesperidoside	C_27_H_30_O_14_	191.20	579.17	10.00 ± 1.96	Flavones/Flavanones
Hesperetin	C_16_H_14_O_6_	769.59	301.07	9.74 ± 0.72	Flavones/Flavanones
Neohesperidin dihydrochalcone	C_28_H_36_O_15_	344.56	595.20	9.14 ± 2.04	Chalcones/Dihydrochalcones
Naringenin	C_15_H_12_O_5_	359.56	271.06	6.96 ± 1.00	
Naringin	C_27_H_32_O_14_	201.50	873.28	6.49 ± 1.24	
Kaempferol-3-O-galactoside	C_21_H_20_O_11_	210.22	449.11	6.33 ± 2.35	Flavonols/Flavanonols
Jaceosidin	C_17_H_14_O_7_	463.67	329.07	6.23 ± 1.40	Flavones/Flavanones
Pinocembrin	C_15_H_12_O_4_	642.39	257.08	5.94 ± 1.65	Flavones/Flavanones
Neohesperidin	C_28_H_34_O_15_	263.85	609.18	5.41 ± 0.92	Flavones/Flavanones
Apigenin 7-O-glucuronide	C_21_H_18_O_11_	913.97	429.08	5.18 ± 1.32	Flavones/Flavanones
Nobiletin	C_21_H_22_O_8_	511.41	385.13	5.03 ± 1.42	Flavones/Flavanones
5,7-Dihydroxy-2-methyl-4H-chromen-4-one	C_10_H_8_O_4_	222.98	191.03	4.59 ± 1.06	Chromones
Isoxanthohumol, (2S)-	C_21_H_22_O_5_	884.50	353.14	3.36 ± 0.91	Flavones/Flavanones
Didymin	C_28_H_34_O_14_	166.00	887.29	2.88 ± 0.52	Flavones/Flavanones
Apigenin 7-O-beta-D-glucoside	C_21_H_20_O_10_	191.20	579.15	1.89 ± 1.22	Flavones/Flavanones
Kaempferol 3-O-glucoside	C_21_H_20_O_11_	182.26	449.11	1.87 ± 0.65	
5,7-Dihydroxychromone	C_9_H_6_O_4_	107.44	177.02	1.66 ± 0.38	Chromones
Procyanidin B2	C_30_H_26_O_12_	189.41	797.19	1.55 ± 0.22	Anthocyanins
Apigenin 7-O-beta-D-glucoside	C_21_H_20_O_10_	219.30	431.10	1.53 ± 0.43	Flavones/Flavanones
Taxifolin	C_15_H_12_O_7_	360.63	287.06	1.47 ± 0.61	
Eriodictyol	C_15_H_12_O_6_	358.89	287.06	1.26 ± 0.28	Flavones/Flavanones
Hispidulin	C_16_H_12_O_6_	445.58	299.06	1.23 ± 0.33	Flavones/Flavanones
Isorhamnetin	C_16_H_12_O_7_	475.60	315.05	1.07 ± 0.48	Flavonols/Flavanonols
Apigenin	C_15_H_10_O_5_	439.69	271.06	1.03 ± 0.18	
Didymin	C_28_H_34_O_14_	344.56	577.19	0.83 ± 0.05	Flavones/Flavanones
Kaempferol	C_15_H_10_O_6_	455.86	287.06	0.22 ± 0.42	
Quercetin 3-beta-D-glucoside	C_21_H_20_O_12_	134.55	463.09	0.08 ± 0.00	
4′,6,7-Trihydroxyisoflavone	C_15_H_10_O_5_	191.20	271.06	0.08 ± 0.00	Isoflavones/Isoflavanones
isorhamnetin-3-O-glucoside	C_22_H_22_O_12_	199.98	477.10	0.02 ± 0.01	Flavonols/Flavanonols

**Figure 1:**
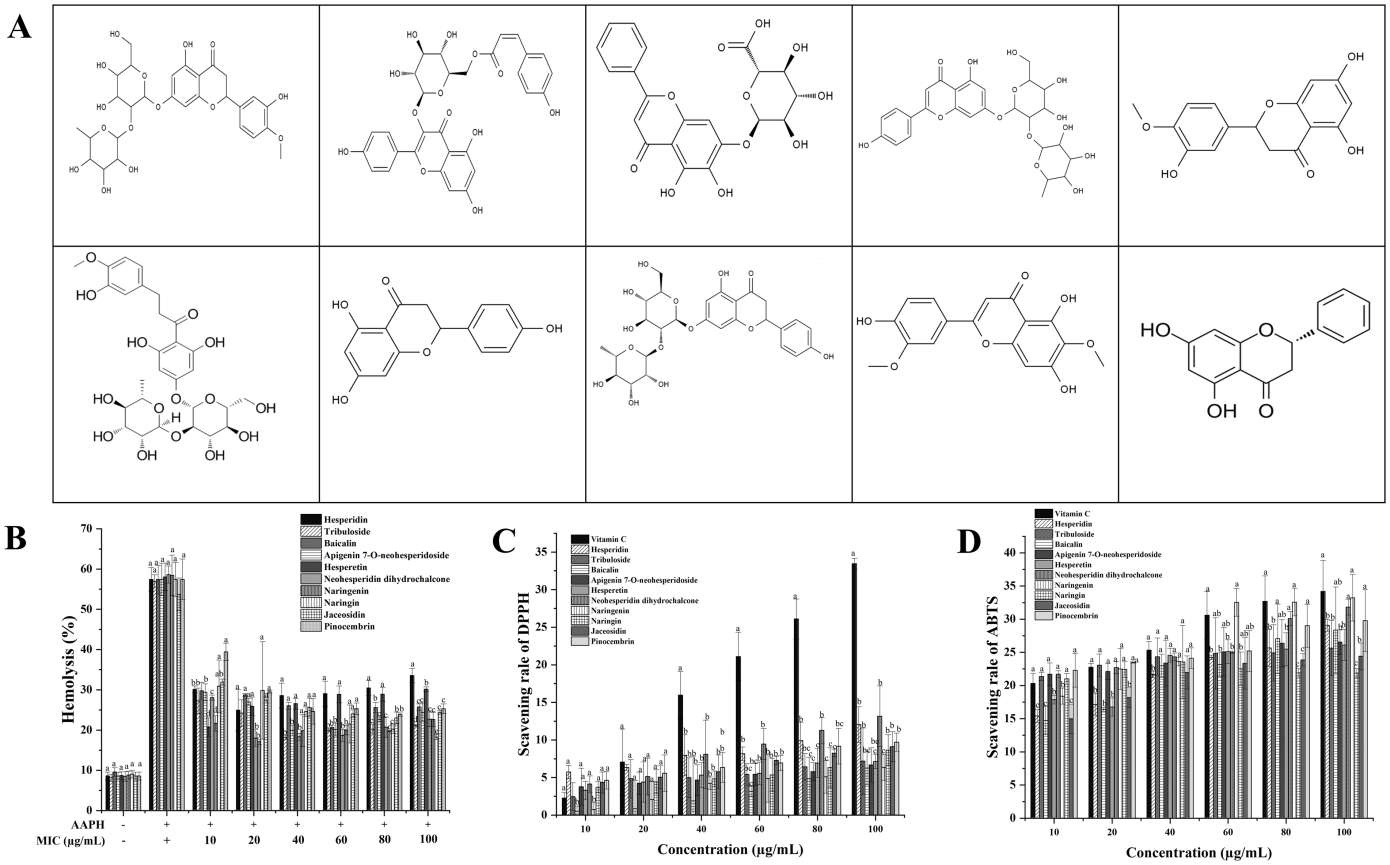
The molecular structures and antioxidant capacities of ten major flavonoids from WPJ. **(A)** Structure of the 10 flavonoid species. **(B)** AAPH-induced the erythrocyte hemolysis activity of the 10 flavonoid species; **(C)** DPPH radical scavenging capacities of 10 flavonoid species; **(D)** ABTS radical scavenging capacities of 10 flavonoid species. Data with different letters indicated significantly different (*p* < 0.05).

The molecular structures and antioxidant capacities of ten major flavonoids are presented in Figure 1. Vitamin C was used as a control to assess the differences in antioxidant capacities among the flavonoids. The inhibition rates of the ABTS and DPPH free radicals increased with the concentrations of the flavonoids in each group (Figures 1B,C). Combined with the composition analysis of the 10 flavonoids, the top four IC_50_ values in the ABTS for naringenin, baicalin, hesperidin, and neohesperidin dihydrochalcone were 184.63 ± 12.32 μg/mL, 234.27 ± 21.36 μg/mL, 240.21 ± 16.87 μg/mL, and 263.44 ± 19.58 μg/mL, respectively. The top four DPPH IC_50_ were neohesperidin dihydrochalcone (468.76 ± 36.76 μg/mL), hesperidin (697.36 ± 57.23 μg/mL), baicalin (774.86 ± 58.94 μg/mL), and naringenin (863.33 ± 48.97 μg/mL). In the AAPH-induced erythrocyte hemolysis assay (Figure 1D), hesperidin, neohesperidin dihydrochalcone, and naringenin demonstrated significant protective effects at a concentration of 20 μg/mL, whereas hesperetin exhibited a notable protective effect at 10 μg/mL (*p* < 0.05). The antioxidant and anti-inflammatory activities of flavonoids are closely linked to their structural skeletons; methoxylation of the flavone −OH groups typically enhance the anti-inflammatory activity of these compounds (43, 44). The structural skeletons of ten flavonoids are integrated, with hesperidin, tribuloside, baicalin, apigenin 7-O-neohesperidoside, hesperetin, naringenin, naringin, jaceosidin, and pinocembrin containing a C-5 bond within their structures. Among them, hesperidin and hesperetin have C-5′ bonds, and apigenin 7-O-neohesperidoside, naringenin, naringin, and jaceosidin have C-4′ bonds. Neohesperidin dihydrochalcone is unique as its antioxidant capacity and anti-inflammatory effects are mainly based on the -OCH_3_ group in its structure (45). Therefore, based on the flavonoid content and the structural skeletons identified through LC–MS analysis, it can be hypothesized that hesperidin, baicalin, neohesperidin dihydrochalcone, and hesperetin would exhibit significant structural activity in terms of antioxidant effects in the WPJ.

### 3.4 Effects of WPJ on LPS-stimulated RAW264.7 cells

**Figure 2:**
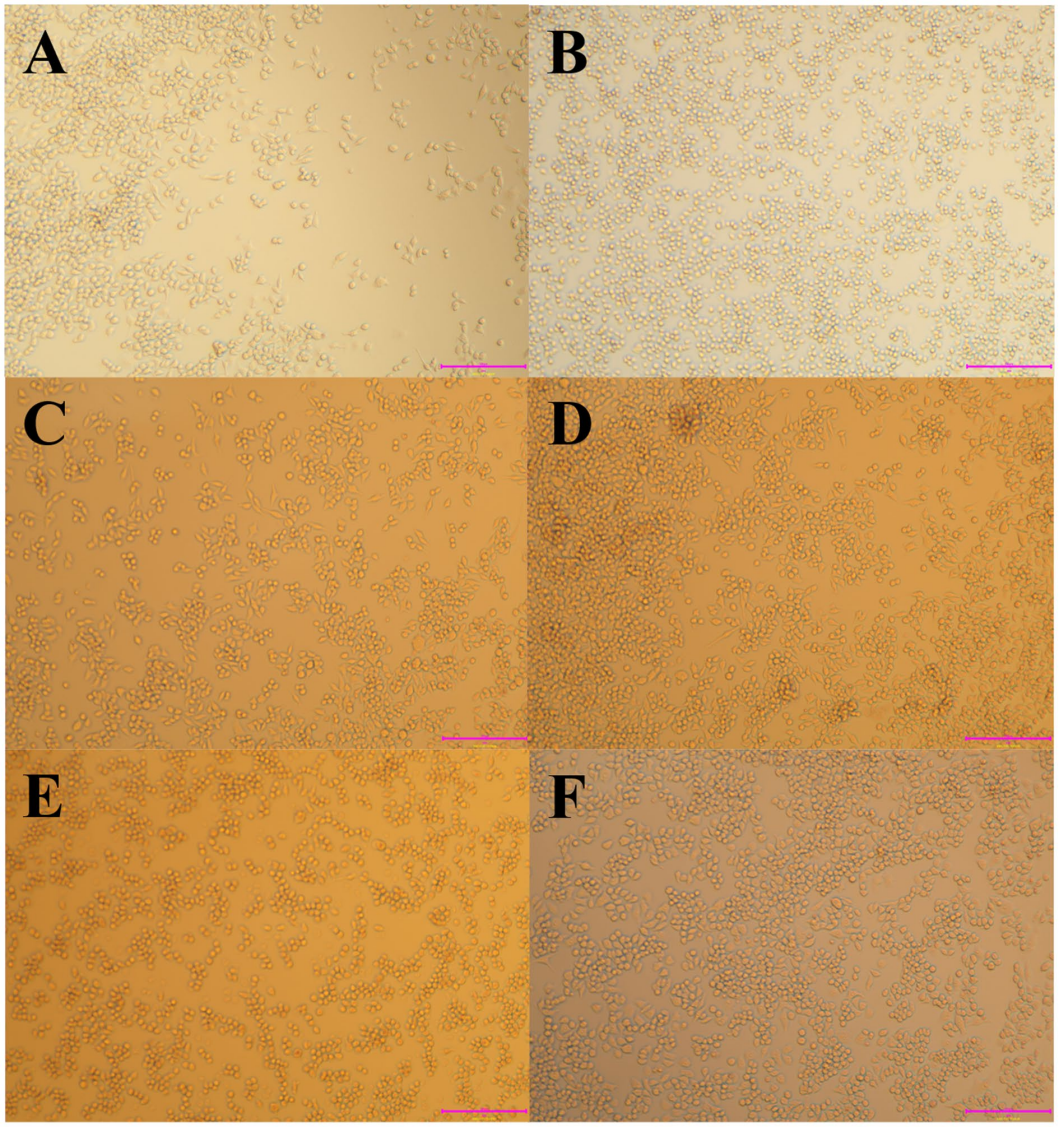
WPJ effects on the morphology of RAW264.7 cells (20×). **(A)** CK, **(B)** Model, **(C)** 25% WPJ, **(D)** 50% WPJ, **(E)** 75% WPJ, and **(F)** KCN. CK, RAW264.7 cells neither treated with WPJ nor stimulated with LPS; Model, RAW264.7 cells subjected to LPS; 25% WPJ, RAW264.7 cells treated with 25% WPJ and stimulated with LPS; 50% WPJ, RAW264.7 cells treated with 50% WPJ and stimulated with LPS; 75% WPJ, RAW264.7 cells treated with 75% WPJ and stimulated with LPS; and KCN, RAW264.7 cells treated with KCN and stimulated with LPS.

**Figure 3:**
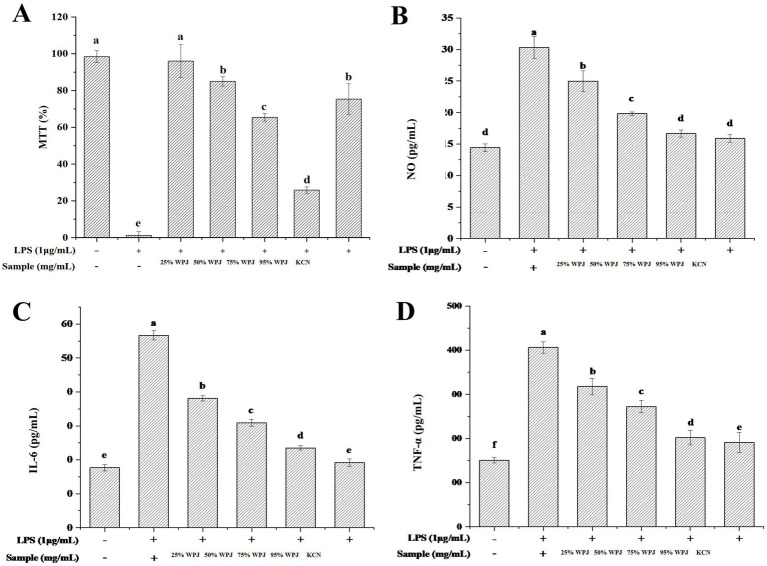
Effects of different WPJ concentrations on cells viability, NO, IL-6 and TNF-α level of AW264.7 cells. **(A)** Effect of WPJ on Cell viability; **(B)** NO production from RAW264.7 cells; **(C)** IL-6 secretion in RAW264.7 cells, and **(D)** TNF-α secretion in RAW264.7 cells. Data with different letters indicated significantly different (*p* < 0.05).

To confirm that WPJ alleviated the LPS-induced cytotoxicity in the RAW264.7 cells, an inverted microscope was used to observe the morphology of the RAW264.7 cells in different treatment groups under 20 × magnification (Figure 2). In the CK group, RAW264.7 cells exhibited a round shape with smooth edges and no pseudopodia (Figure 2A), while the LPS-treated group (model group) displayed significant morphological alterations. The morphological features of the LPS-treated group indicated macrophage activation, characterized by increased cell size and the extension of pseudopodia from one or both sides of the cells (Figure 2B). However, RAW264.7 cells pre-treated with either WPJ or KCN prior to LPS stimulation exhibited morphology similar to that of the CK group (Figures 2C–F), suggesting that both WPJ and KCN help maintain normal cellular morphology and suppress macrophage activation in LPS-stimulated RAW264.7 cells. Furthermore, the results of the cell viability assay, assessed using the MTT method, are presented in Figure 3A. A 25% concentration of WPJ did not significantly affect the viability of RAW 264.7 cells stimulated with 1 μg/mL LPS. Increasing the WPJ concentration to 50–75% resulted in a decrease in cell viability from 85.01 ± 2.56% to 65.42 ± 2.03%. At a WPJ concentration of 95%, cell viability was significantly reduced to 25.86 ± 1.75% compared with the CK group (*p* < 0.05). Consequently, WPJ concentrations of 25, 50, and 75% were selected to represent low, medium, and high dosages, respectively, for subsequent experiments. These findings suggested that WPJ exerts effects comparable to those of KCN in alleviating LPS-induced cytotoxicity.

To investigate the anti-inflammatory effects of WPJ, LPS was used to stimulate the release of NO, IL-6, and TNF-*α* in RAW 264.7 macrophage cells, thereby mimicking a chronic inflammatory environment. When LPS and WPJ acted together for 4 h, different concentrations of WPJ had significant inhibitory effects on the NO secretion (Figure 3B) (*p < 0.05*). As previous results, LPS can promote the release of a large number of cytokines, such as TNF-α and IL-6 on the cell surface, leading to the infiltration of inflammatory cells (20). However, co-treatment with LPS and WPJ resulted in a significant, dose-dependent reduction in cytokine levels across all groups (*p* < 0.05), indicating that a specific concentration of WPJ effectively inhibited the LPS-induced inflammatory response in RAW264.7 cells. The WPJ treatment also significantly reduced the levels of the inflammatory factors IL-6 (Figure 3C) and TNF-α (Figure 3D). This is consistent with the results of Javier et al. regarding the anti-inflammatory activity of flavonoids extracted from pomelo *in vitro* (46).

### 3.5 Histological analysis of the WPJ effects on lung inflammation on model mice

**Figure 4:**
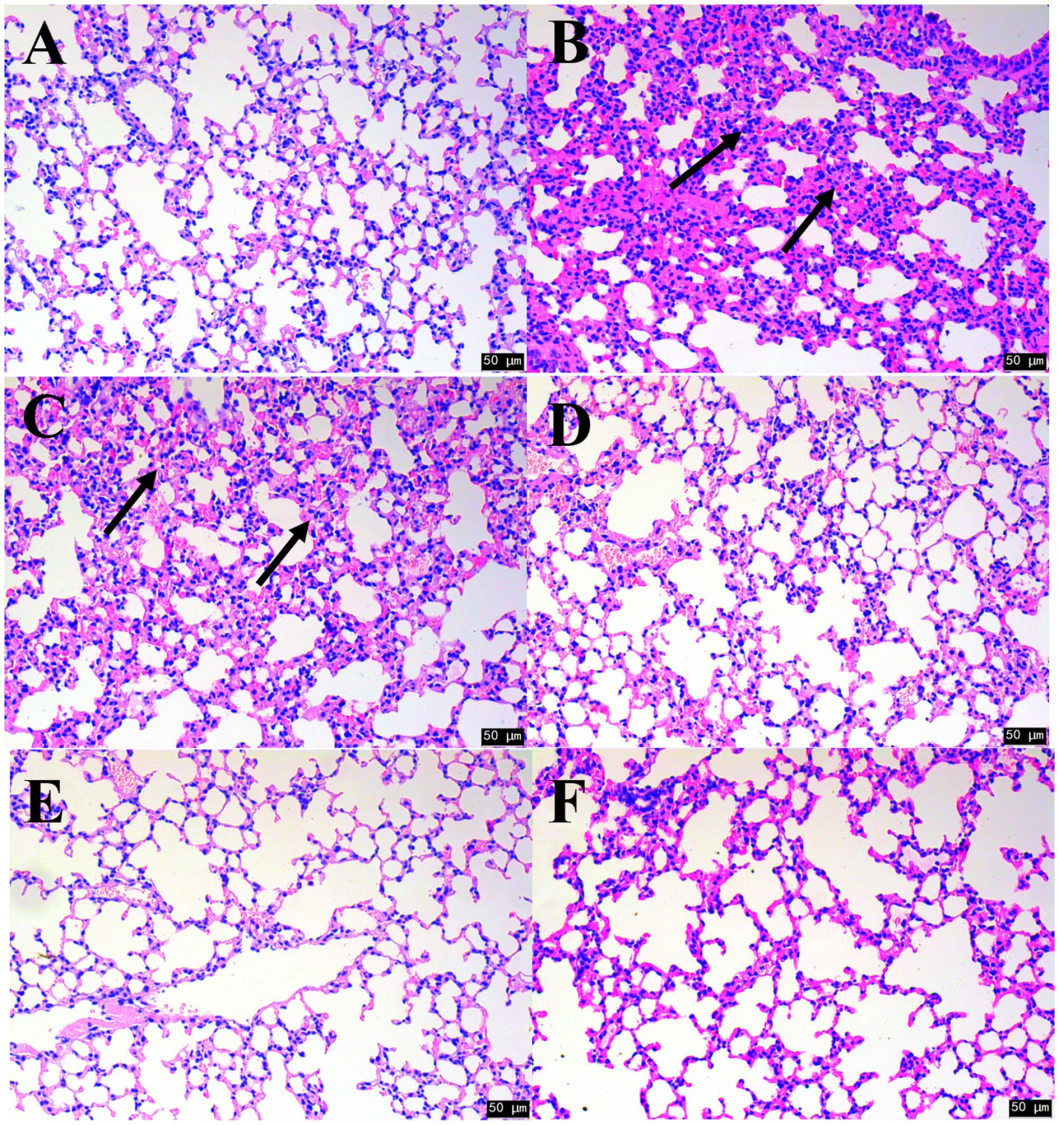
H&E staining in the mouse lung tissues from different groups (100×). **(A)** CK; **(B)** Model; **(C)** 25% WPJ, **(D)** 50% WPJ, **(E)** 75% WPJ and **(F)** KCN. CK, mice samples neither treated with WPJ nor NH₄OH; Model, mice samples treated with NH₄OH; 25% WPJ, mice samples treated with 25% WPJ and NH₄OH; 50% WPJ, mice samples treated with 50% WPJ and NH₄OH; 75% WPJ, mice samples treated with 75% WPJ and NH₄OH; and KCN, mice samples treated with KCN and NH4OH. Data with different letters indicated significantly different (*p* < 0.05).

To evaluate the WPJ effects on lung, we develop the model mice with phlegm turbidity and lung obstruction. The histological analysis of lungs showed that the mice in the CK group showed intact bronchial mucosal epithelium, neatly arranged cilia, no obvious inflammatory cell exudation, and intact alveolar structures without obvious expansion (Figure 4A). In the model group, the bronchi of the mic were significantly thickened, their goblet cells were increased, inflammatory cells had infiltrated the mucosal layer, and some alveoli fused into pulmonary bullae, indicating that sputum turbidity and inflammation had occurred in the mice (Figure 4B) (47). This result proved that a mouse model of sputum turbidity-induced lung disease had been successfully established (48).

The efficacy of WPJ in alleviating cough and reducing sputum production in model mice was evaluated through histopathological observations for each experimental group. As the result, in model mice treated with WPJ, bronchial wall thickening was reduced, goblet cell numbers decreased, and less inflammation cells were observed along with the increasing of WPJ concentration (Figures 4C–E). In addition, model groups treated with 50 and 70% WPJ exhibited morphologies similar to those of the model group (model mice treated with KCN), suggesting that WPJ could significantly improve pathological damage to lung cells and their structure, while also reducing the infiltration of inflammatory cells into the lung interstitium. In particular, when the WPJ concentration was 75%, the effect was similar to that of KCN, which was close to that of the CK group. The similar results also observed in the function study of Shiwei Longdanhua formula, the formula used for treating respiratory diseases by inactivating the globlet cells and decrease the secretion of gel forming mucins in the LPS-mediated model (49) (Figure 4F).

### 3.6 Expectorant and antitussive effect of WPJ

**Figure 5:**
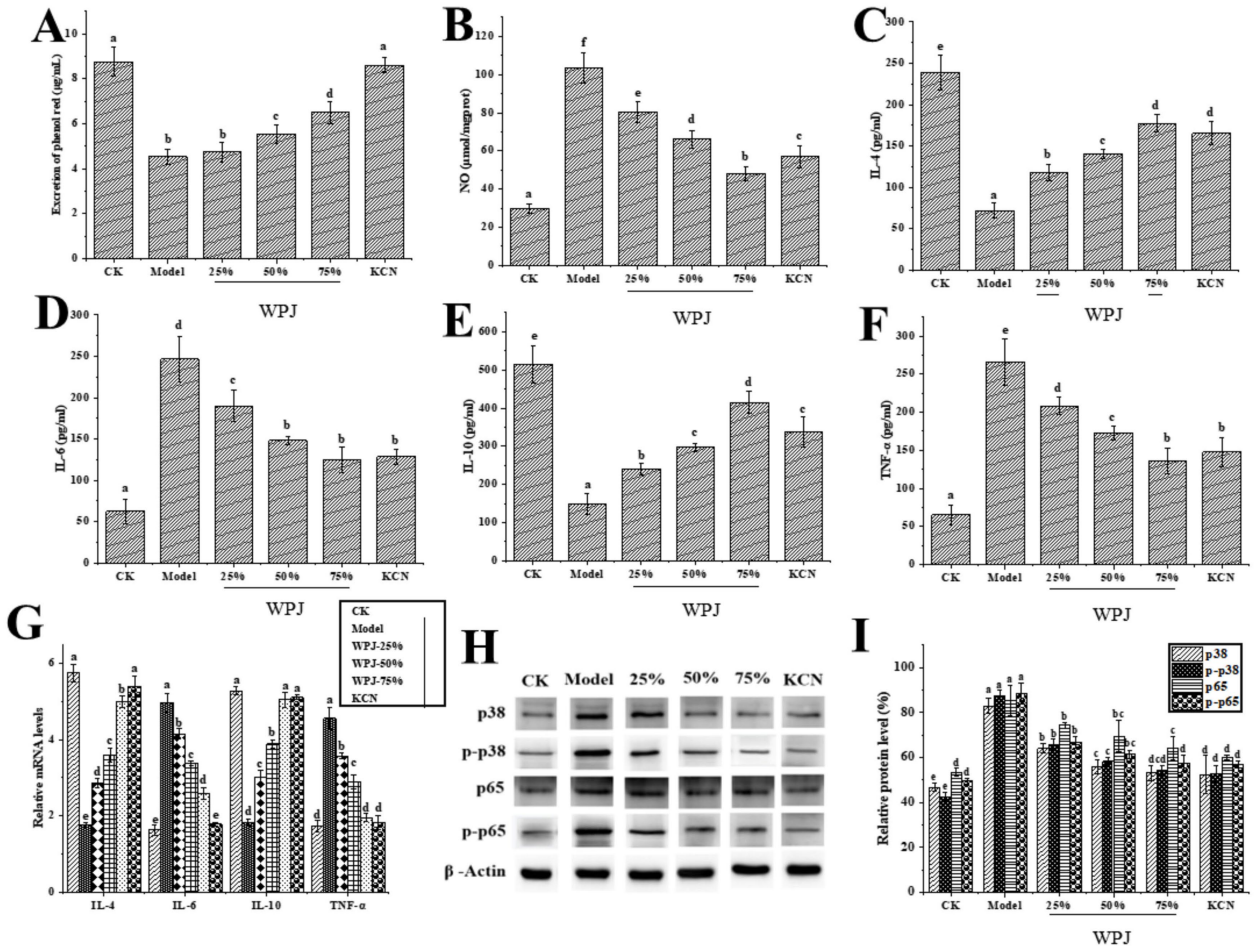
**(A)** Effects of the WPJ treatment on the phenol red secretion in mice. **(B–G)** Inflammatory levels and gene expression of the IL-4, IL-6, IL-10, and TNF-α in the mouse lung tissue. **(H,I)** Expression and phosphorylation of p38 and p65 proteins in the mouse lung tissue. CK, mice samples neither treated with WPJ nor NH₄OH; Model, mice samples treated with NH₄OH; 25% WPJ, mice samples treated with 25% WPJ and NH₄OH; 50% WPJ, mice samples treated with 50% WPJ and NH₄OH; 75% WPJ, mice samples treated with 75% WPJ and NH₄OH; and KCN, mice samples treated with KCN and NH_4_OH. Data with different letters indicated significantly different (*p* < 0.05).

The expectorant and antitussive effects of WPJ were compared with those of the CK, and the results are presented in Figure 5A and Table 3. In the expectorant assay, 75% WPJ significantly increased phenol red secretion in a dose-dependent manner, which increase from 4.37 to 43.84%. In the antitussive assay, WPJ inhibited cough in a dose-dependent manner, and this inhibition increase from 30.00 to 55.00%, with the concentration from 25 to 75%. Though KCN showed more potent expectorant and antitussive effect (90.87% phenol red secretion rate and 70.00% cough inhibition) than WPJ, WPJ still showed the ability on cough inhibition. The expectorant and antitussive effects of WPJ are most likely due to their major flavonoids. Flavonoid, and phenolic derivatives are the main components of the pomelo. Original WPJ contains 950.68 ± 7.65 mg/100 g of a complex mixture of flavonoids (Table 1), and the expectorant activity of flavonoids had been thought to be mediated by inhibiting oxidative and reductive processes and decrease the activity of cholinesterase and xanthinoxidase (50). The major flavonoids including hesperidin, hesperetin, naringenin and naringin of WPJ showed potential for cough inhibition. For example, naringenin could significantly increase the secretion of phenol red from mouse tracheas and enhance the basal lysozyme secretion. Treatment with naringenin could inhibit the LPS-induced mucin increase (51). Moreover, Seyedrezazadeh’s study indicated that co-treatment of hesperetin and naringenin could significantly decreased subepithelial fibrosis, smooth muscle hypertrophy in airways, and lung atelectasis (52). Besides, the other flavonoids such as tribuloside, neohesperidin and baicalin were reported play important role on inflammation treatment indicating they may work on cough inhibition (52–55).

**Table 3:** Effect of WPJ on phenol red secretion and cough inhibition.

Group	Increase of phenol red secretion (%)	Cough inhibition (%)
CK	94.59	–
Model	–	100 ± 0.00
25% WPJ	4.37 ± 2.67	30.00 ± 3.54
50% WPJ	22.29 ± 1.92	40.00 ± 2.36
75% WPJ	43.84 ± 4.55	55.00 ± 5.24
KCN	90.87 ± 5.47	70.00 ± 6.21

CK, mice samples neither treated with WPJ nor NH₄OH; Model, mice samples treated with NH₄OH; 25% WPJ, mice samples treated with 25% WPJ and NH₄OH; 50%WPJ, mice samples treated with 50% WPJ and NH₄OH; 75% WPJ, mice samples treated with 75% WPJ and NH₄OH; and KCN, mice samples treated with KCN and NH₄OH. Data with different letters indicated significantly different (*p* < 0.05).

### 3.7 Effects of the WPJ on the number of inflammatory cells from model mice

Moreover, the blood cell analysis was performed on model mice. Blood cell analysis revealed significant differences in the number of inflammatory cells among the CK and experimental groups. Compared with the CK group, the number of white blood cells and lymphocytes (neutrophils, lymphocytes, monocytes, eosinophils, and basophils) in the model group was significantly higher (*p < 0.05*), indicating the inflammatory effects was observed in the model group (56). However, compared with the model group, the number of inflammatory cells in the WPJ-treated group was significantly lower (*p <* 0.05), suggesting that WPJ may alleviate pulmonary inflammation in mice (Table 3).

### 3.8 Effects of WPJ on the MAPK pathway

Coughing is a complex biological process modulated by multiple factors, involving a variety of theories and mechanisms, where inflammation response is a basic feature. Previous research indicated that antitussive effects may be regarded as related to its anti-inflammatory properties via the improvement of mucociliary clearance and the reduction of chemokines from epithelial cells (57). To verify the association between the antitussive and anti-inflammatory effects of the WPJ, the lungs of the mice were compared with those of the CK group. As Figure 5, the levels of NO (Figure 5B), IL-4 (Figure 5C), IL-6 (Figure 5D), IL-10 (Figure 5E), and TNF-*α* (Figure 5F) in the alveolar lavage fluid of the model group were significantly higher than those of the CK group (*p* < 0.05). To further investigate the protective effects of WPJ on inflammatory cell activity in mice with phlegm-turbid lungs, mRNA transcription levels were measured. As shown in Figure 5G, varying doses of WPJ inhibited the transcription levels of pro-inflammatory cytokines IL-6 and TNF-α, while significantly upregulated the transcription of anti-inflammatory cytokines IL-4 and IL-10, thereby influencing the protein expression levels of these cytokines. These results suggested that WPJ may reduce inflammatory and enhance the immune response in mice (Table 4).

**Table 4:** Blood routine inflammatory cell count.

Measurements	Group	Count	Reference values	Units
White blood cells	CK	4.20 ± 0.93^b^	4–10	×10^^9^/L
	Model	6.91 ± 1.25^a^		
	25% WPJ	3.32 ± 0.56^c^		
	50% WPJ	3.17 ± 0.43^c^		
	75% WPJ	4.49 ± 1.26^b^		
	KCN	4.11 ± 0.44^b^		
Neutrophils	CK	2.55 ± 0.40^c^	2–7	×10^^9^/L
	Model	5.61 ± 1.57^a^		
	25% WPJ	3.01 ± 0.27^b^		
	50% WPJ	2.07 ± 0.26^c^		
	75% WPJ	2.10 ± 0.69^c^		
	KCN	2.51 ± 0.55^c^		
Lymphocytes	CK	0.95 ± 0.82^c^	0.8–4	×10^^9^/L
	Model	3.96 ± 0.11^a^		
	25% WPJ	2.26 ± 0.28^b^		
	50% WPJ	1.99 ± 0.42^b^		
	75% WPJ	2.32 ± 0.69^b^		
	KCN	1.36 ± 0.13^c^		
Monocytes	CK	0.16 ± 0.36^b^	0.12–1.2	×10^^9^/L
	Model	1.15 ± 0.36^a^		
	25% WPJ	0.26 ± 0.02^b^		
	50% WPJ	0.19 ± 0.27^b^		
	75% WPJ	0.14 ± 0.02^b^		
	KCN	0.22 ± 0.31^b^		
Eosinophils	CK	0.01 ± 0.02^b^	0–0.5	×10^^9^/L
	Model	0.09 ± 0.02^a^		
	25% WPJ	0.03 ± 0.00^b^		
	50% WPJ	0.01 ± 0.01^b^		
	75% WPJ	0.02 ± 0.01^b^		
	KCN	0.01 ± 0.01^b^		
Basophilic	CK	0.03 ± 0.02^b^	0–0.1	×10^^9^/L
	Model	0.07 ± 0.01^a^		
	25% WPJ	0.01 ± 0.01^b^		
	50% WPJ	0.01 ± 0.00^b^		
	75% WPJ	0.01 ± 0.02^b^		
	KCN	0.01 ± 0.00^b^		

CK, mice samples neither treated with WPJ nor NH₄OH; Model, mice samples treated with NH₄OH; 25% WPJ, mice samples treated with 25% WPJ and NH₄OH; 50%WPJ, mice samples treated with 50% WPJ and NH₄OH; 75% WPJ, mice samples treated with 75% WPJ and NH₄OH; and KCN, mice samples treated with KCN and NH₄OH. Data with different letters indicated significantly different (*p* < 0.05).

Previous studies showed that the activation of IL-6 by TNF-α is known to be mediated through the p38 mitogen activated protein kinase (MAPK) pathway (58). And activation of the MAPK pathway can promote the phosphorylation of key proteins in the NF-κB signaling pathway, thereby activating the NF-κB signaling pathway. Therefore, the expression levels of the MAPK p38 and NF-κB p65 proteins, along with their phosphorylated forms, were assessed and found to be closely associated with the inflammatory response. Compared with the CK group, the expression and phosphorylation levels of p38/p65 pathway proteins in the lung tissue of mice in the model group were significantly increased (*p* < 0.05) (Figures 5H,I). In contrast, treatment with various concentrations of WPJ significantly reduced the expression and phosphorylation of p38/p65 pathway proteins compared to the model group (*p* < 0.05). Several studies have shown that flavonoids in plants can relieve cough and reduce phlegm (59, 60). We thus inferred that the flavonoids in WPJ had anti-inflammatory effects on LPS-induced lung inflammation in RWA.264.7 cells and mice. They may also inhibit the phosphorylation of p38, p65, and other proteins, and inhibit the activation of related signaling pathways (61, 62), thereby regulating the mRNA transcription of inflammatory factors, and ultimately playing a role in the regulation of inflammation (7, 63).

Coughing facilitates the expulsion of mucus, microbes, and foreign particles from the respiratory tract, thereby serving as a protective mechanism against pulmonary infection and inflammation (64). However, suppressing inflammation may ease a cough. In respiratory systems, the flavonoids show spasmolytic activity. Antiphlogistic and antiallergic effect of flavonoids is enhanced by concomitant administration of vitamin C (75). Quercetin, pinocembrin, possesses significant bacteriostatic effect to gram-positive as well as gram-negative bacteria (76). Ramnezin, fizetin, and related antocyans inhibit the growth and replication of tuberculous bacilli. Most of flavonoids mark out by significant antioxidant action (77). All of mentioned flavonoid properties together with antitussive-expectorant activity participate probably in positive and beneficial effect of drugs such as *Plantago lanceolata*, *Malva sylvestris*, *Polygonium aviculare*, *Primula veris*, *Verbascum densiflorum*, and others in the therapy of respiratory tract diseases. WPJ is rich in nutrients, including flavonoids, vitamin C, and vitamin E which are predominant in its anti-inflammatory and antioxidant activities. Vitamins C (65) and E (66) are important antioxidants with anti-inflammatory and immune system enhancement features, and consequently, they provide protection against inflammation. The number and positions of the hydroxyl groups in flavonoids is another crucial factor for anti-inflammatory inhibition (67). Chae al. showed that flavonoids and their derivatives inhibit inflammation through the MAPK pathway and inhibit the expression of the inflammatory factors IL-6 and IL-10, thereby preventing inflammation in the lungs (20). Flavonoids such as naringenin, baicalin, hesperidin, and neohesperidin dihydrochalcone demonstrated protective effects against liver inflammation by inhibiting NF-κB activation and modulating inflammatory mediators (68–71). We have hypothesized that the synergistic action of various active substances in the WPJ regulates the expression of inflammatory genes in the lungs of mice, thereby achieving antitussive and expectorant effects.

## 4 Conclusion

In the present study, we enzymatically hydrolyzed pomelo after juicing, thereby determined the bioactivity of the WPJ expression after 1 year of storage. WPJ can reduce the inflammatory response induced by LPS in RAW264.7 cells and a mouse model of sputum turbidity lung resistance, mainly by inhibiting the activation of MAPK and NF-κB signaling pathways, to reduce the inflammatory effects. Based on previous research, we have developed an oral liquid that relieve coughing and reduce phlegm. This work confirms the pharmacological potential of WPJ for respiratory diseases, demonstrating its expectorant and antitussive action. Further studies should be performed to evaluate the mechanisms of action involved.

## Data Availability

The original contributions presented in the study are included in the article/Supplementary material, further inquiries can be directed to the corresponding authors.
