# Supplementary material for: Evaluation of flavonoid composition and biological activities of hydrolyzed whole pomelo juice
Source: Front Nutr. 2026 Jan 7;12:1730735. doi: 10.3389/fnut.2025.1730735 (PMC12819249; doi:10.3389/fnut.2025.1730735)
Supplement: Supplementary file 1 [file Data_Sheet_1.pdf]

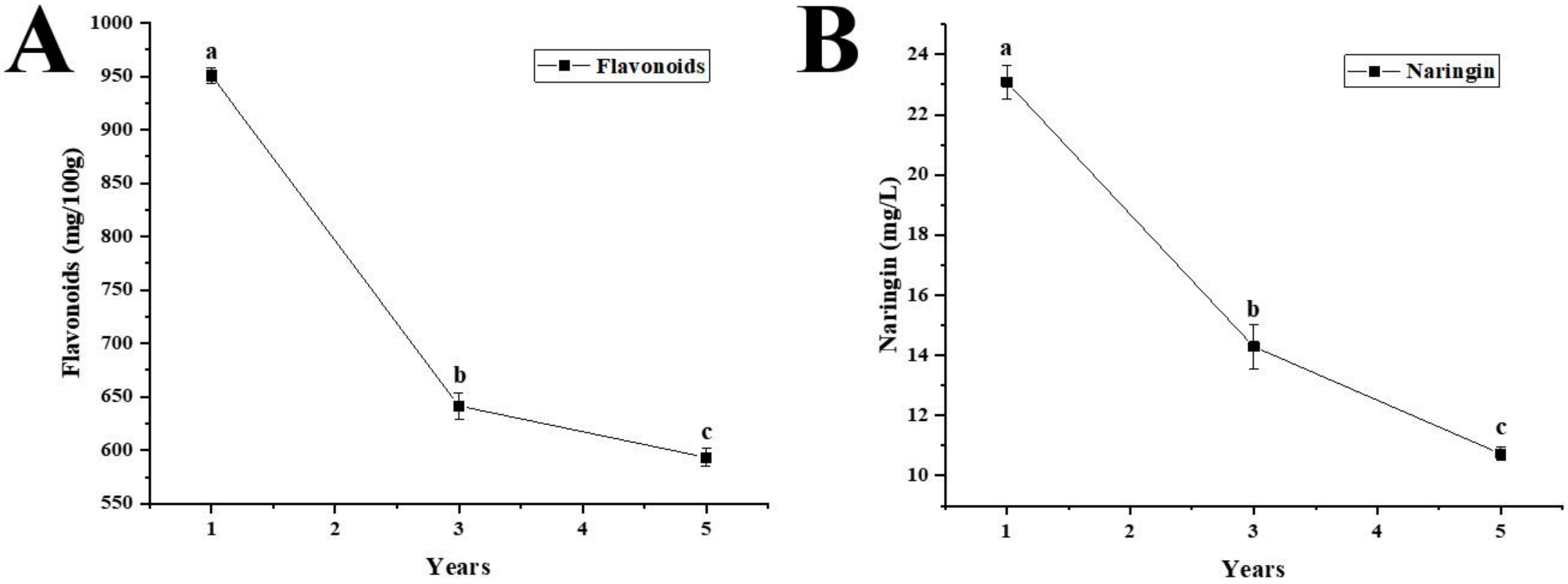

Figure S1 Figure 1. Effect of storage time on the total flavonoid and naringin contents.

(**A**) Total flavonoid content of WPJ; (**B**) Naringin content in the WPJ. Data with different letters indicated significantly different *($p < 0.05$)*.

Table S1 Other nutritional composition of WPJ

Nutrients	Content
Protein (g/100g)	0.82±0.01
Pectin (g/100g)	1.07±0.01
Reducing sugar (g/100g)	0.89±0.02
Ash (g/100g)	0.13±0.01
Essential oils (g/100g)	1.23±0.21
Insoluble fiber (g/100g)	0.79±0.02
Soluble fiber (g/100g)	0.19±0.02
Total fiber (g/100g)	0.98±0.01
Water content (g/100g)	84.48±0.32
Carbohydrate (g/100g)	13.26±0.23
Fats (g/100g)	0.32±0.02
